# Book Reviews

**Published:** 1827-11

**Authors:** 


### CRITICAL ANALYSES.

Quæ laudanda forent, et quæ culpanda, vicissim

Illa, prius, cretâ; mox hæc, carbone, *notamus*.—PERSIUS.

*A Treatise on the Nature and Cure of Rheumatism; with Observations on Rheumatic Neuralgia, and on Spasmodic Neuralgia, or Tic Douloureux.* By CHARLES SCUDAMORE, M.D. F.R.S. Honorary Member of Trinity College, Dublin; Physician in ordinary to his Royal Highness the Prince Leopold of Saxe Coburg, &c. &c.—8vo. Longman and Co. London, 1827.

WE have been rather free in our remarks on former productions of Dr. SCUDAMORE's, because we have thought they bore the stamp of precipitation, while there did not appear to us to be any thing of sufficient novelty or importance in their contents to justify their hasty publication. With regard to the present work, however, we find that our author looks upon it as due to the public by a former promise, as well as in the discharge of an obligation of a different kind ; he adopts as his motto the words of LA BRUYERE—" Je rends au public ce qu'il m'a prêté; j'ai emprunté de lui la matière de cet ouvrage; il est juste que l'ayant achevé avec toute l'attention pour la vérité dont je suis capable, et qu'il mérite de moi, je lui en fasse la réstitution."

The volume before us unquestionably has had more labour bestowed upon it than any other of the author's works, except that on Gout, of which it is the counterpart. It is extended, but not complete ; minute, but not always precise. Of 576 pages, to which it extends, not less than 333 (considerably more than one-half,) are devoted to cases, which, being given in the same large type as the text, swell out the bulk greatly beyond what was necessary. As it professes to be a " systematic treatise," it commences with a detail of the opinions of various writers, from Hippocrates downwards, all of which we shall pass over, and come to what belongs to Dr. Scudamore himself. His definition of rheumatism, and description of its varieties, are as follow :

" Pain of a peculiar kind, usually attended with inflammatory action, affecting the white fibrous textures belonging to muscles and joints, such as tendons, aponeuroses, and ligaments; the synovial membranes of the bursæ and tendons; and nerves; occasioned by the influence of variable temperature, or by direct cold, or by moisture.

"The species of rheumatism are two, acute and chronic; the former of which may be divided into the acute and sub-acute.

"The Acute Rheumatism.—Pain, with inflammation of the ligaments of the joints, and usually those of the larger joints; or of tendons and aponeuroses; of the sheaths of tendons; of the bursal membranes; and of nerves; aggravated by motion; for the most part attended with external redness of a bright colour, and with fever which has exacerbations, and sometimes distinct remissions; with copious partial perspirations, commonly of an acid odour; and high-coloured urine, depositing abundantly lateritious sediment.

"Sub-acute Rheumatism.—As the term implies, this form of the complaint is intermediate in its symptoms between the high constitutional fever which belongs to the acute rheumatism, and the absence of fever which is characteristic of the chronic. It is much more solitary than the acute, and it often happens that the bursal texture is alone affected, and especially in the knee; but any of the other textures just described, and also branches of nerves, may be affected with partial inflammation in such a degree as not to disturb the constitution with much fever.

"The Chronic Rheumatism.—Pains affecting the ligamentous and tendinous textures, synovial membranes, and nerves, without external signs of inflammation, and unaccompanied by fever, but aggravated by motion." (P. 11.)

After these divisions, our author proceeds to make some general observations on rheumatism, in the course of which we are informed that the ligaments, tendons, and the tendinous parts of muscles and aponeurotic expansions, together with the bursæ mucosæ, are the structures most frequently affected by the disease. Nerves are also said to be often affected; but "it does not happen as a common occurrence that in the large joints the synovial membrane is affected by rheumatic inflammation; and, when it does happen, it is more by extension of the disease from the parts nearer to the surface than primarily." In this statement we cannot concur: we have seen so many cases in which the knee and shoulder joints have been affected with a circumscribed swelling, evidently produced by the capsular membrane being distended by fluid, with very little or no affection of the neighbouring parts, that we look upon it both as a common occurrence and primary disease. Throughout the volume we perceive a tendency, on the part of the author, to confound those forms of rheumatism which appear to us to be so distinct from each other; he states, indeed, (page 27,) "that rheumatism receives considerable modification of symptoms, and also requires a corresponding modification of treatment, from the influence of the particular texture which may be affected;" but we do not think that his general directions or particular prescriptions, as given in his cases, are in keeping with this observation.

Into the description of the symptoms of rheumatism, which is full and satisfactory, it would be tedious and unnecessary for us to enter. Among the consequences of the disease he enumerates the affection of the heart, and correctly remarks that it does not usually take place from the retrocession of rheumatism, but that "the rheumatic action continues in almost the same force in the limbs, and rarely, if ever, wholly subsides." It certainly has been too common to look upon rheumatism of the heart as an example of metastasis; at the same time we think Dr. Scudamore has himself fallen into error in ranking it among the *consequences* of acute rheumatism, seeing it is only one of its most severe and exquisite forms. The dura mater may also become the seat of inflammation associated with rheumatism, but no attempt is made to point out the particular modifications of the disease which gives a liability to the one rather than the other of these internal affections. According to Dr. CHAMBERS,* rheumatism of the fibrous membranes exclusively, or almost exclusively, gives rise to affections of the heart, and rheumatism of synovial membranes to affections of the encephalon; and, in conformity with this opinion, we observe that in the only case of this last description given by Dr. Scudamore (p. 264), the synovial textures are stated to have been affected.

Among the changes of structure, suppuration is certainly the most uncommon, and, although our author has alluded to two instances of this nature, we confess that they do not appear to us to be very satisfactory.

"When I was at Paris, I was informed by M. Breschet of a case of acute rheumatism which proved fatal; and, as many unusual symptoms had arisen in the course of the disease, an inspection after death was made. In and about every affected joint there was found a considerable quantity of purulent secretion. This case furnishes an exception to the general rule, that rheumatic inflammation does not terminate in suppuration." (P. 34.)

From this case, as the particulars are not given, and as "many unusual symptoms" had occurred, it is obvious that no conclusions can well be deduced. The only other case we have been able to find in which suppuration took place, is at page 289: it had obviously nothing whatever to do with rheumatism, and is headed "inflammation of a doubtful

* See cases published in this Journal for July and August, 1826.

nature at the knee-joint." We do not mean to assert that rheumatism never produces suppuration of the parts, but merely that our author has not, so far as we can see, furnished us with any example of it.

We pass by the causes, predisposing, exciting, and proximate, which are severally discussed, to arrive at the treatment.

*Of bleeding.*—A general idea of Dr. Scudamore's opinions with regard to this remedy may be gathered from the following extract:

"General bleeding in this disease is a remedy of great importance; but its employment requires much consideration and judgment. If it be proper in the particular case, it is far more advantageous to resort to it promptly, than to allow delay; because symptoms increase, and constitutional power lessens, with the progress of disease.

"It may be laid down as a principle, that, as relating to the local inflammatory action merely, this is not the agent in which we should place our confidence; for it disappoints our expectation of relieving the pain of the disease, unless as the pain and the local inflammation may be connected with the true inflammatory diathesis. In no way is a degeneracy into chronic symptoms so certainly introduced, as by that injudicious employment of general bleeding, which enfeebles the constitution, and still leaves the rheumatic disposition in great force: nor does the articular inflammation itself yield to the use of general bleeding in the manner which we might expect.

"If, however, a patient of strong muscular fibre, and of the sanguineous temperament, be seized with acute rheumatism, in full health, an immediate and free use of the lancet is indispensably necessary. The depletion is to be repeated until the hardness and fulness of the pulse become reduced to a state of softness and moderation." (P. 69.)

These directions are in accordance with the views of the most experienced practitioners, and we have nothing to object to them; but the paragraph which immediately follows contains what appears to us a very unnecessary refinement, and one of very questionable accuracy: we mean the advice of M. Laennec to judge of the propriety of blood-letting by the stethoscope rather than by the pulse, as the former "furnishes a rule more sure than the tact of the most able practitioners." Dr. Scudamore, indeed, observes, that he has, "in some critical circumstances, derived this particular aid from the use of the stethoscope;" but it really appears to us to be carrying the matter rather too far to advise that, in a case of rheumatism or inflammation of any part not within the chest, we should apply the stethoscope in order to discover whether the patient will bear further depletion or not, instead of feeling the patient's pulse as heretofore. Our author, however, is but half a convert to mediate auscultation, for he thinks that "we may in general trust to the experienced touch."

*Emetics*.—Of these, little need be said.

"If the patient be seized with threatening symptoms of an acute attack, shortly after some convivial occasion, on which he has indulged freely and improperly, the administration of an emetic will, in all probability, be highly useful. But, should inflammatory action be urgently present, it should be abated by the use of the lancet before we have recourse to full vomiting." (P. 90.)

*Cathartics and diuretics*.—We do not exactly perceive why these are placed under one head; the more, as we find it to contain observations on various medicines belonging to neither denomination,—such as tartarised antimony, opium, hyoscyamus, sulphate of quina, and mercury. These observations are desultory, but not uninteresting.

"I have been much satisfied with the effects of a draught composed of the carbonate of magnesia, carbonate of potash, sulphate of magnesia in small doses, tartarised antimony, lemon juice in fit proportion to neutralise the carbonate of potash, and the acetum colchici, with some agreeable distilled water and syrup. The draught may be taken in effervescence, or otherwise. The addition of the tartar emetic is exceedingly valuable; for my increasing experience with this medicine convinces me that it is one of the most useful remedies which we can employ for the removal of inflammatory action; and, in proportion as we employ it with judgment, so do we diminish the necessity of using the lancet. Its influence is much more permanent than that of digitalis; which, although on many occasions to be regarded as a most valuable auxiliary in the treatment of inflammation, is yet liable to the objection of its restraining action rather than subduing inflammation, and masking rather than curing the disease.

"Upon the first administration of the tartar emetic, it usually sickens to the degree of causing vomiting; but this effect is useful, and it is surprising how quickly the stomach accommodates itself to this medicine, so that it ceases even to nauseate. The maximum and minimum doses of the tartar emetic, which I usually employ in the combination just mentioned, are one grain and one-eighth of a grain; and of the acetum colchici, a drachm and a half and half a drachm.

"In concurrence with this draught, I prescribe calomel with the compound extract of colocynth, at night, for the purpose of producing a more free excretion of bile than would be effected merely by the saline aperient. It also assists in reducing the inflammatory diathesis, and increases the action of the absorbent system. The range of dose which I prefer is from one grain to five, and I am not aware of any superior advantage from employing larger doses. In urgent cases of acute rheumatism, I continue the use of calomel until the gum becomes slightly affected.

"It will usually be expedient to add to the pills just mentioned a portion of opium, or a dose of extract of poppy, or of hyosciamus, in order to delay the action of the bowels, and assist repose. It may be convenient to join with these ingredients a full dose of opium, for the purpose of relieving or preventing pain. If we are not making use of tartarised antimony in the medicine which we give at regular intervals, it will be beneficial to add it, or James's powder, to the pills. The omission of the compound extract of colocynth may be sometimes advisable.

"In regard to the freedom and continuance of this treatment, we shall inform ourselves, in great measure, by a regular observation of the nature of the excretions, alvine and urinary; for, while the fæces are unnaturally dark, and the urine is dense, of a deep colour, turbid, or even depositing lateritious or pink sediment, the fluid portion being clear, it is incumbent upon us to make daily employment of purgative medicine. When the excretions acquire a natural appearance, the acute symptoms of inflammation usually subside, and then our active treatment must be exchanged for the occasional use of a sufficient quantity of an aperient for the regulation of the bowels; at the same time taking advantage of the absence of fever to introduce the trial of tonic medicine and restorative diet.

"When the stomach is in too irritable a state to allow the continuance of the draught just mentioned, it will be expedient to give, in the morning early, a mixture with senna, salts, and manna, rendered palatable by the addition of some aromatic water, &c. Subsequently, at regular intervals, saline medicines will be useful.

"Such, according to my experience, is the decided advantage of following up a course of purgative or aperient and alterative treatment, when it is not forbidden by any remarkable debility of the constitution. When this exists, a different method of treatment must be adopted. Should there be sufficient remission of fever, it would be important to administer the sulphate of quinine, or some form of bark; but of this practice I shall speak under its proper head.

"To the above exception to the perseverance in the active purgative treatment, I may add, that when the delicacy of constitution, which I have mentioned, is attended by a continuance of febrile irritation, which militates against the employment of any preparation of bark, we shall find it expedient to be contented with the moderate measures of using saline antimonial medicines and sedatives, and the application of leeches for the relief of particular parts which may be affected with severe pain.

"Even in these circumstances, we shall usually derive advantage from the effects of a mild alterative every night, or on alternate nights, consisting of a grain or half a grain of calomel, a grain of James's powder, with a small portion of crude opium, or four grains of extract of poppy, giving some mild aperient in the morning; or, under some circumstances, directing a lavement. If the bowels be easily irritated, four or five grains of the hydrarg. c. cretâ, or pilul. hydrargyri, will be preferable to the use of calomel.

"As a rule of practice, I hold it to be very important in the treatment of acute rheumatism, carefully to avoid producing mercurial fever, which tends to aggravate rather than relieve the rheumatic symptoms, and produces a new state of distress scarcely inferior in its sufferings to those of the original disease." (P. 92.)

*Tartar emetic.*—In addition to the remarks on this excellent medicine contained in the above paragraph, we have a separate article upon it, which consists almost exclusively of quotations from the published opinions of Laennec, with regard to the use of antimony in inflammation, and which we therefore think it out of place to notice here.

*Sudorifics.*—Tartarised antimony again.

*Narcotics and sedatives.*—Of these opium is unquestionably the best. Our author speaks favourably of it, but alludes to certain injurious effects, which he believes will scarcely ever be met with, if it be given in combination with calomel, and followed by brisk purging.

"The use of opium, as the most important of our sedative medicines, is an eminent point of consideration in our study to relieve the intense sufferings of acute rheumatism. An active state of inflammatory diathesis must be removed by the means already detailed, before we can with any propriety employ opium with freedom, if at all. I have seen permanent delirium, and serious aggravation of fever, produced by the premature administration of opium. It indeed usually happens that opium does not even relieve pain when given during any considerable state of inflammatory action; and this will always be found an important point in practice.

"A due attention must also be paid to the functions of the bowels, the kidneys, and the skin, in order that we may be enabled to seek the advantages which opium is often so happily capable of affording. No contra-indication, therefore, being present, we are to decide upon the particular preparation, and its combination with other ingredients. Most commonly the severest pain takes place during the night, and this also is the period at which it is most desirable to interpose the aid of narcotics; for nature becomes the more exhausted if repose be denied at the season when most wanted and expected. But, if all the indications of treatment are correctly fulfilled, we may also, with every propriety, direct an opiate dose at any time during the day, when pain is urgent. I can recommend, from much satisfactory experience, the following mixture:

"℞. Potassæ Carbonat. gr. cviij.; Succ. Citric. (recentis) ℥ij.; Mist. Camphoræ ℥iijss.; Liquoris Opii Sedativ. ʒjss. ad ʒij.; Syrup. Tolutan. ℥ss.; Antimon. Tartarisat. gr. j. ad gr. ij. M. fiat mistura.

"Of this mixture, one, two, or three tablespoonsful should first be taken, according to the degree of pain; and a dose should be repeated every hour or two, till relief is obtained. I may observe of opium, that the acetate of morphine is the least stimulating preparation, and that, given in a saline draught with camphor mixture and a small portion of hydrocyanic acid, it will sometimes succeed better than any other form, relieving pain effectually, and not causing headache or confusion. The dose of the acetate is from a quarter of a grain to a grain; and of hydrocyanic acid in this mixture, from one to two drops; to be repeated as the occasion may require.

"The effects of opium are, in a very remarkable degree, dependent upon the influence of pain; so that persons who are exceedingly incommoded by even a very small dose, under ordinary circumstances, can bear without inconvenience a large quantity when suffering much pain. There is this charity in disease, that it allows a free use of its appropriate remedy.

"With some few individuals, however, so completely does the nervous system refuse to accommodate itself to the influence of opium, that we are compelled to resort to the trial of weaker narcotics. In some cases, and especially when the inflammatory diathesis does not prevail, and general rheumatic pain, and consequent irritation, predominate, the following draught will be found beneficial:

"℞. Liquoris Ammon. Acetat. ℥ss.; Vini Colchici m. xx. ad ʒss.; Syrupi Papavaris ʒj.; Mist. Camphoræ ℥j. M. fiat haustus sexta vel octava quâque horà sumendus.

"It has appeared to me that the wine of the seeds of colchicum is milder than the wine of the roots, but that the latter can be more relied upon for its efficacy. It will occasionally be advisable to prescribe this kind of draught at bedtime only. If it happen that the use of colchicum is not suitable in any form, we may be led to the choice of the compound powder of ipecacuanha in a saline draught, giving it with more or less frequency, as the symptoms may require.

"The weaker narcotics, as henbane, conium, and poppy, cannot be relied upon for the relief of acute pain, although they may be competent to relieve moderate pain, and more especially to allay irritation and restlessness; but, with this latter view, I have more confidence in the properties of the genuine lactucarium, or lettuce milk, which may be given in a dose of from three to six grains. Stramonium (prepared from the seeds) was strongly recommended by the late Dr. Marcet, as an occasional substitute for opium. In my experience, it has not produced such satisfactory results as one or other form of opium joined with antimony; but, in other parts of this treatise, I shall have occasion to speak more particularly of this medicine.

"With some patients, labouring under acute rheumatism, every kind of narcotic proves unfavourable in its effects; and saline antimonial medicines, with mild aperients and occasional alteratives, alone succeed; joining, probably, the influence of general or local bleeding, or both. The inconvenience attending the use of opiates, to which I have just alluded, may arise from two causes: peculiarity in the constitution of the patient, which forbids the influence of any narcotic medicine; or the countervailing irritation which arises from the inflammatory diathesis. In the former difficulty, I have often seen the best effects produced by the administration of a lavement with tincture of opium and a few ounces of water, in quantity from twenty to ninety minims. Of the means to be used for removing the inflammatory diathesis, I have already treated." (P. 104.)

*Nitre.*—Dr. Scudamore, in one instance of sub-acute rheumatism, knew a patient take half an ounce of nitre daily: "some benefit was derived;" but he considers the practice as much inferior to the other modes detailed.

*Peruvian bark.*—On this he observes—

"I have, on several occasions, made trial of it, observing the rules laid down by Haygarth; but very generally I have found that it has aggravated both fever and pain. I know from experience that cases do now and then occur, in which the remission of fever is so distinct, and the true inflammatory diathesis is so entirely absent, that it becomes highly proper to make trial of this treatment; but, in such circumstances, I am led to prefer the use of the sulphate of quinine, with or without the addition of sulphate of magnesia, as the state of the alimentary canal may require. It should be given in a dose of two grains, at short intervals. The encouragement to its use will be, that the tongue appears moist, the pulse soft, the skin relaxed, the urine not of a deep colour, and without very copious sediment. The doubtful part of the question, it will be seen, relates to the propriety of using bark during the height of the acute symptoms; for its value at the moment of convalescence is well confirmed by experience. But neither this nor any one point in practice can be reduced to strict rule." (P. 115.)

We think even more unfavourably than our author of cinchona in *acute* rheumatism: he says that its value at the moment of convalescence is well confirmed by experience; but we greatly question this assertion, for we have frequently seen patients beginning to recover from acute rheumatism, who immediately suffered a relapse on bark being ordered by way of hastening their convalescence.

*Regimen and diet.*—Keeping the patient too warm is reprobated; "comfortable" covering is recommended; and the "lightest" diet enjoined.

*Local treatment.*—Leeches are the only local means recommended in the acute stage of the disease; but, as soon as the fever subsides, and the pains become fixed in particular parts, we are told that "much advantage will be derived from the use of a lotion, composed of two parts of alcohol and one of mistura camphoræ, applied tepid, by means of several layers of linen, and over them a piece of oil-skin, just extending beyond the linen, but not used as a complete envelope, which might cause the part to be heated. The oil-silk prevents the speedy drying of the rags, and renders the evaporation slow; which, in rheumatism, appears to be more useful than if effected quickly. At night, it is often advantageous to apply a tepid poultice, prepared with this lotion and equal parts of grated bread and linseed meal."

*The convalescence.*—According to our author, the recovery will be much assisted by various preparations of bark:—this was said before under the head "Peruvian bark," and we have there already expressed our doubts of its accuracy. Exercise and friction are advised; and the necessity of overcoming the "seeming incapacity of motion" which remains after an acute attack of rheumatism, is pointed out.

The next subject connected with acute rheumatism is *metastasis,* particularly the affection of the heart commonly attributed to this cause. The existence of this disease is as well made out as any other pathological fact, and the numerous references to different writers given by our author are therefore unnecessary, so far as regards its establishment, while there is little in the description of the symptoms which is not generally known. We pass on, therefore, to the treatment, and dwell upon this the more because we do not agree in opinion with Dr. Scudamore on this part of the subject.

"On the immediate occurrence of the symptoms, a copious bleeding from the arm is imperatively demanded; and the blood should be allowed to flow until the pulse become soft, or until syncope be produced. It must be repeated accordingly as it is called for by returning symptoms. Twenty or thirty leeches should be applied over the region of the heart, followed by fomentation and poultice; but cupping, if it could be borne, would usually be preferable. As soon as the active symptoms of inflammation become relieved, a large blister should be applied over the left side of the chest." (P. 139.)

The point of practice to which we object in this quotation is that which regards the quantity of blood to be drawn. We look upon it as very dangerous advice to let the blood flow "*until syncope be produced.*" We would just reverse this, and say that the risk of syncope ought never to be incurred. Small bleedings only are admissible, (say twelve to fourteen ounces;) but these must be repeated till the symptoms yield, at such intervals as can be done without the risk of fainting. We know that in the diseased condition of the heart its functions are more easily interrupted than under ordinary circumstances, and that it has happened that syncope being produced in the disease in question by a large bleeding, the heart has never regained its power, and the patient has thus died from the remedy rather than the disease. In this very case the patient had experienced great relief from a moderate bleeding, and it was argued that, if the loss of twelve ounces produced a certain quantum of benefit, the loss of twenty-four would produce still more.

"We must seek to moderate the action of the heart by the use of appropriate medicines, and the most efficacious will be found in the tartar emetic and in digitalis, which may be given in a saline draught, every two or three hours, in full doses." (P. 139.)

Tartar emetic may be of use, but we would not trust to it; —digitalis we believe to be entirely useless under such circumstances: its first effect in the majority of cases, is to increase the heart's action, which is not in fulfilment of any rational indication.

"As this dangerous form of complaint comes on in the midst of a disease which has been already treated, it is not probable that the bowels will be in a loaded state; and bodily quietude is so important, that the action of purgative medicine should be avoided during the height of the symptoms. Small doses of calomel, repeated at intervals of six or eight hours, will, under most circumstances, assist the cure. Extract of poppy, or extract of henbane, will be a useful addition to the calomel; for it is of importance to lull irritability while we are using active means to reduce inflammatory action." (P. 139.)

There can be no question of the propriety of emptying the bowels; and, important as "bodily quietude" certainly is, we doubt whether it need ever be so much disturbed by purgatives as to warrant their omission. Calomel is spoken of as a very secondary remedy, and as to be administered in "small doses:" we think that this medicine, in the affection of the heart under consideration, is next in importance to blood-letting, if indeed it be secondary to it: it is as much indicated here as in iritis, and for precisely the same reason, —viz. to prevent the deposition or promote the removal of coagulable lymph. It ought, therefore, to be administered in full and frequently-repeated doses, so as to bring the system under its action as speedily as possible. Extracts of poppy and henbane are recommended to lull irritability:—why not opium? We have seen a considerable number of cases of rheumatic affection of the heart in which opium (combined with calomel) has been freely administered, and have been impressed with a very favourable opinion of its utility.

"The limbs, and especially the rheumatic parts, should be fomented with flannels wrung out of hot water, having the addition of spirits and vinegar, and afterwards should be wrapped in flannel. Blisters applied to the limbs will certainly prove useful. Sometimes the prompt action of sinapisms will be highly appropriate." (P. 140.)

In this paragraph Dr. Scudamore evidently writes under the impression of the disease being metastasis, although this is contrary to his own, and we believe to the correct, pathology.

"It is important to observe, that, as soon as we have succeeded in reducing the pulse to softness and moderation, we should refrain from further general bleeding; for the rheumatic action appears to diminish the power of the heart very remarkably, and renders it morbidly irritable in a high degree.

"If the disease pass into the chronic form, the symptoms must be treated according to the character which they assume. I shall hereafter show, by some interesting cases, that the irritable state of the heart, which gives rise to palpitation and high action, rather than strength of pulse, is often more successfully treated by the use of tonics, as the sulphate of quinine or the subcarbonate of iron, and a supporting but not an exciting diet, than by debilitating sedatives and severe regimen." (P. 140.)

In this we entirely concur, as may be seen from our remarks on the first passage in which blood-letting is less cautiously recommended. The general impression resulting from a careful perusal of this part of Dr. Scudamore's work certainly is, that he is not fully aware of the danger of syncope under such circumstances, or of the advantages of mercury: thus we are told that, in the subject of Case XV. (by the by, we believe the only instance of affection of the heart from acute rheumatism in the book,) "the poor youth died, notwithstanding the most prompt and active treatment," yet neither mercury nor opium are mentioned. "Upon examination, the fibrous layer of the pericardium was found partially adhering to the heart by recently-formed portions of fibrine, and the bag of the pericardium contained a few ounces of turbid serum." We never saw such a case do well permanently, when the system was not brought under the influence of mercury.

These remarks conclude an account of the first part of the volume before us,—viz. that relating to acute rheumatism. The consideration of the sub-acute and chronic forms of the disease follow; but we do not find any thing in these requiring particular notice. The treatment of the *sub-acute* is similar to that of the *acute*, modified by its comparative mildness; while in *chronic* rheumatism "we may take our choice between a combination of volatile tincture of guaiacum joined with the acetate of ammonia and Dover's powder, or vinum colchici joined with the latter medicines: very full doses of these remedies at bedtime, and smaller doses in the day, should it be expedient to prescribe such repetition." Peruvian bark and sarsaparilla are spoken of as occasionally useful, and local stimulants, baths, and fomentations recommended.

The volume concludes with some observations on *neuralgic affections*, which, however, are not of sufficient interest for quotation.

---

*Observations on the Surgical Pathology of the Larynx and Trachea, chiefly with a View to illustrate the Affections of those Organs which may require the Operation of Bronchotomy: including Remarks on Croup, Cynanche Laryngea, foreign Bodies in the Windpipe, Wounds, &c. &c.* By Wm. Henry Porter, A.M. Member of the Royal College of Surgeons in Ireland; Surgeon to the Meath Hospital and County Dublin Infirmary, and to the Dublin General Dispensary; and Lecturer on Anatomy and Surgery in the Medico-Chirurgical School, Park-street.—8vo. pp. 283. Hodges and M'Arthur, Dublin, 1826.

Whatever may be the subject to which an author has paid particular attention, and to the investigation of which he has devoted his labours, it is naturally considered by him of much interest. In the preface, then, to most works, and in that of the volume now before us amongst the number, we find the importance of the subject duly insisted upon, and the necessity of our paying especial attention to it earnestly pointed out by the author. Mr. Porter, however, cannot be accused of attaching consequence to a class of diseases, which are so trifling in their nature, or so simple in their treatment, as not to require the closest and most attentive inquiry. He is perfectly justified in prefacing his work by the observation, that—

"There are few subjects found to present themselves under more interesting features, either to the surgeon or the patient, than those diseases which interfere with the actions of the respiratory organs. Whoever has in himself experienced the slightest difficulty of breathing, or has witnessed its effects on others, must be aware of the intense anxiety that such diseased action creates:

and when we recollect that in no case can the surgeon more completely or more decidedly display the efficacy of his art,—that he is sometimes enabled to restore the patient from a state of fearful distress to one of comparative tranquillity,—and that, in many instances, he may suddenly snatch him from the jaws of inevitable destruction, we shall not hesitate to acknowledge the importance that attaches itself to this part of surgical science." (P. i.)

Having admitted, then, the fair claim which the author has to the merit of a happy selection of his subject, we proceed to lay before our readers an account of the manner in which he has treated it.

The operation of bronchotomy, and the diseases which may render it necessary, have frequently attracted the notice of professional writers, and several valuable papers have at different times been offered to the public upon the subject. But amongst these Mr. Porter finds no attempt to arrange those diseases in pathological order, to point out the morbid appearances that are discoverable by dissection, and to connect these with each particular symptom. The great aim of all seems to be to inculcate the necessity of resorting to the operation of bronchotomy, and to show that several of those who have been suffered to perish in a miserable state of suffocation might have been thus preserved. But, even if the operation be had recourse to, success is far from certain, and an investigation into the causes of these failures ought to be interesting, as every unsuccessful operation tends more or less to diminish the confidence of the public in professional skill, and, if the real nature of the case be not understood, to introduce timidity and indecision into the mind of the practitioner himself. However cautiously we would withhold our countenance from the premature and unnecessary performance of operations, we are satisfied, with Mr. Porter, that bronchotomy is too seldom practised, and that, when it is performed, it is generally at a period of disease when its success is impossible.

Mr. Porter introduces his subject with some brief yet pertinent observations upon the inflammation of mucous membranes in general. These remarks bear upon the immediate subject; for, as the larynx and trachea have an internal covering of mucous membrane, inflammation of those parts must be governed in a great degree by the laws which appertain to inflammatory affections of a similar structure in other parts of the body. The symptoms which arise, and the effects produced, will of course be greatly modified by the important functions these parts have to perform. Amongst other instructive observations upon the subject of inflammation of mucous membranes, Mr. Porter particularly remarks, that "it sometimes happens that lymph is found affixed on an inflamed mucous surface; but, *excepting in cases occurring under the age of puberty*, it is very doubtful whether this substance, considered as the product of *acute* inflammation, be coagulated lymph or inspissated mucus." We are not aware that this fact (if such it be) has been before pointed out; neither can we vouch for its correctness. In more chronic affections, membranous layers are often formed, and these our author does not deny to be lymph.

"As, for instance, in the bronchial cells, where, from taking the shape of the parts in which they are deposited, they appear ramified like the branches of blood-vessels, and were of old supposed to be portions of the pulmonary artery. These are called bronchial polypi, and are generally found in patients of an advanced age. But the best example of lymph being produced by chronic inflammation is to be found in dysenteria tubulosa, where whole rings of a considerable length come away, and this so frequently, and to such an extent, as to give rise on some occasions to a supposition that they were large portions of the mucous membrane reduced to a state of slough, and thrown off by the efforts of nature." (P. 10.)

In the child, however, there can be no doubt that lymph is the genuine product of active mucous inflammation, "although it would appear that the inflammation occasioning it is of some peculiar or specific character, and confined in this particular effect to the larynx and trachea alone."

The reason why children should be chiefly subject to disease terminating in this manner, has not, we believe, been hitherto explained; but we would observe, that false membranes may be found in the air passages without the occurrence of croup. For example, M. Nysten published a case, in 1815, of poisoning by the inhalation of ammonia during a paroxysm of epilepsy, the principal effect of which was great inflammation of the mucous membrane of the larynx and trachea, and the formation of a false membrane in the larynx and bronchia.* Mr. Porter remarks, that in hooping-cough "there is good reason to believe" that the mucous membrane of the larynx is more or less inflamed, "and yet rarely an adventitious membrane is formed." As far as we can judge from our own experience, there is always in hooping-cough more or less inflammation of the larynx, and we do not apprehend that a

* Bulletin de la Faculté de Médecine de Paris, No. 5.

false membrane is ever formed in this disease. In an Inaugural Dissertation published by a German physician of the name of SCHMIDT, there are some experiments to prove that artificial inflammation, excited in the windpipe of animals, *only produces the adventitious membrane in those which are very young*. In croup, the lymph is sometimes expelled by coughing, and a few cases have thus terminated favourably, when such a result was the least to be expected. "But, on the other hand, this loose and floating substance has been thrown by the violence of the cough against the rima glottidis, become entangled there, and caused the death of the patient by instantaneous suffocation, at a period of the disease and under circumstances otherwise promising a speedy recovery."

In a public discussion some months ago upon this subject, in which we took a part, this fact was denied by several practitioners, who refused to admit that death was ever thus caused in cases of croup. We have, however, no hesitation in agreeing with the opinion of Mr. Porter. And the fact is not unimportant, inasmuch as it would lead us to be careful of giving a decidedly favourable prognosis, although the severity of the disease might be greatly diminished.

Another common occurrence, alluded to as connected with inflammation of mucous surfaces, is hemorrhage without any apparent abrasion or lesion of the membrane. We have seen three or four cases of this kind, in which large quantities of blood have been thrown from the stomach by vomiting, and also passed by stool. Upon dissection, the whole mucous surface of the stomach and intestines presented a dark and pulpy appearance, but no lesion or abrasion could be discovered. The patients, to whose cases we refer, were all much advanced in life, and of debilitated constitution. It appeared as if the capillary extremities had lost their power, and had consequently permitted a large flow of blood to take place.

We have always been of opinion, and our practice has been modified accordingly, that—

"In connexion with the subject of mucous inflammation, spasm constitutes too prominent a symptom to be passed over in silence; for, although it is so far not necessarily connected with the inflammatory action that it may be, and often is, present without it, yet, on the other hand, it so certainly manifests itself on every occasion where inflammation appears, adding considerably to the patient's sufferings, and very frequently causing his dissolution, that it may well be considered as forming an important symptom in affections of this nature. Spasm, however, is not a disease either of mucous membrane or of the submucous tissue; for neither of these structures appear to possess within themselves a power of contractility adequate to explain the phenomena of this irregular action; but of the muscles situated around or in the neighbourhood of mucous canals, the natural and healthy uses of which are connected with the functions of them. In proof of which it may be observed that spasm does not occur in the membranous mucous ducts, or in that part of the urethra anterior to the bulb; whilst it is most frequent in those situations where the canals are subjected to the influence of strong, frequent, and irregular actions of adjoining muscles, as in the bulbous portion of the urethra and in the larynx. Whatever can excite these muscles to action will occasionally be the cause of spasm. Sometimes it appears spontaneously, or at least its occurrence will not admit of explanation. Sometimes it would seem to be a sympathetic affection, as exemplified in the spasmodic croupy respiration of children, whilst under the irritation of teething or of deranged digestion; and sometimes it is the result of a more direct stimulus applied either to the mucous membrane or the muscles themselves." (P. 24.)

Whatever may be the exciting cause of spasm, its effects with reference to the respiratory organs are dreadful; in the child, particularly in the infant, they are sometimes suddenly and unexpectedly fatal. Even in the adult state, Mr. Porter is aware of a case in which death could be accounted for in no other manner. It need scarcely be added, then, that in such cases we should employ antispasmodics in conjunction with our anti-inflammatory remedies.

Having given a slight sketch of the principal pathological facts connected with the inflammation of mucous membranes, Mr. Porter proceeds to the discussion of his more immediate subject, which could not advantageously have been undertaken without these introductory remarks; "for, however the respiratory tube may be affected, or in whatever structure the disease may have been originally situated, still the affection of the membrane that lines it will exercise such a decidedly modifying influence on its symptoms that they can neither be understood nor explained without some previous acquaintance with the pathology of mucous tissues."

Besides the numerous accidents to which the respiratory tube is exposed by its situation and peculiar functions, it is also liable to many idiopathic diseases, which, varying in some respects, will still resemble each other in all the symptoms occasioned by simple interruption or derangement of respiration. Mr. Porter does not enter into the consideration of many of these diseases. He confines himself to the surgical department of the subject; "to those accidents and diseases for the relief of which the operation of bronchotomy has been either practised or proposed."

Falling within this description, according to our author, are "idiopathic affections of the larynx and trachea, acute inflammation of the mucous membrane occurring in the child, constituting croup or cynanche trachealis;—spasmodic croup without the existence of inflammation;—inflammation of the submucous tissue of the larynx in the adult, or acute cynanche laryngea;—thickening of the mucous membrane, or chronic cynanche laryngea;—alteration of structure in the laryngeal cartilages, or phthisis laryngea;—sloughing and death of the cartilages;—and the pressure exercised by abscess or tumor in the neighbourhood of the windpipe, obstructing the passage of the air." (P. 27.)

We believe that cases of "spasmodic croup without the existence of inflammation" are sometimes fatal to infants; but we apprehend that in such instances the attack is generally so very sudden, so entirely unexpected, and so speedily destructive, that the operation of bronchotomy can rarely, if ever, be performed in time to save the patient.

The author gives a very correct practical view of the nature and treatment of croup; and, with respect to bronchotomy, he observes that there must be great difficulty in distinguishing the cases in which the operation is likely to be attended with success; for very frequently the inflammation commences in the bronchial cells, and proceeds upwards in the windpipe.

"This is an affection in which an operation could not possibly be of service, and there is no mode of distinguishing accurately as to what has been the original seat of the disease. This one consideration must involve every case in obscurity, and render the success of an operation a matter more dependent on chance than on judgment." (P. 48.)

At the latter stages of croup, it would be absurd to think that an operation could possibly prove beneficial,—

"Unless it could be supposed that a wound of the windpipe could remove cerebral congestion; and therefore, whenever convulsions have occurred, or that the patient appears comatose or sinking, let no man undertake it as a last resource; for it is a resource that will avail him little, and, after his patient's death, he may esteem himself fortunate if a great part of the blame is not laid to the account of himself and his knife.

"It will be necessary to draw a distinction between the struggles a patient may make to free himself from some obstruction in the windpipe and the convulsions just alluded to; for in both cases the patient's countenance will become dark and purple, his eye suffused and staring, and his respiration heaving and laboured; and both these affections occur at a late period of the disease. If the inflammation is about to subside, and to terminate in an extensive effusion of mucus, the patient's struggles to relieve himself from the oppression naturally created by the accumulation of fluid in the respiratory tube, must be not only distressing but injurious, and may prove fatal. In this case an operation, which will afford a clear and easy passage for the fluid, will probably be followed by the happiest consequences; or if there was reason to believe that, on the total subsidence of the inflammation, the membranous lymph existed in the trachea purely in the condition of a foreign body, an opening which would afford means for its easy expulsion might also be judicious. But it is unfortunate that no symptoms exist, by which a practitioner can positively establish the propriety of his operation: there are in both these forms of disease the same cough,—the same difficult respiration,—the same struggles to inflate the lungs, and to free the air passages from the impediments that are within them. To these sources of difficulty may be added, that any obstruction to the air cannot endure long without the lungs becoming loaded and oppressed, and therefore if the operation is not performed almost at the exact minute between the subsidence of the inflammation and the commencement of the effusion, it will scarcely be successful. Thus it happens that, in the great majority of cases, when bronchotomy has been performed, the patient finds himself wonderfully relieved; great quantities of mucus are expelled by the wound; respiration is free; and a surprising degree of tranquillity seems to be suddenly obtained. But the calm is only deceitful. In the course of two or three days the patient begins to sink. He is unable to expectorate the mucus, which now accumulates in greater abundance than before. He becomes heavy and languid; is with difficulty roused from this state of stupor, and generally within four or five days after the operation he dies comatose." (P. 50.)

The author does not, however, assert positively that there are no cases of croup that might not be benefited by the operation; but he asks,

"What is the most favourable time for adopting it? and what are the symptoms which will regulate the surgeon's practice? Can any man exactly draw a line of distinction between the varieties of croup, and say that the inflammation in one case had commenced in the bronchial cells, and in another at the larynx;—that in one instance the adventitious membrane had been formed, and in another it had not? Can he distinguish between chronic bronchitis and morbid thickening of the laryngeal membrane? And, if he cannot, is not his operation altogether empirical,—just as likely to work out evil as good,—undertaken without principle where it may do injury, and perhaps abandoned where it might have proved beneficial?" (P. 63.)

Mr. Porter contends, that if it were possible to place a list of those cases in which bronchotomy had not proved serviceable, in array against those wherein it had seemed to be useful, it would scarcely be necessary to advance any farther argument in proof of its uncertainty; and medical men would seek rather to improve their internal treatment, than trust to an operation from which experience holds out such slender hopes of benefit.

Several cases are given to illustrate the pathological varieties generally to be met with in the examination of croupy subjects. In two, the operation of bronchotomy was performed: they terminated fatally.

In our opinion, Mr. Porter has not taken too unfavourable a view of the operation of bronchotomy in cases of croup. That it may afford temporary relief in some cases, we can easily believe; nor do we doubt that a few rare instances have occurred in which it has saved the patient. But still the insurmountable difficulty of distinguishing the particular case, or stage of the complaint, in which it can be presumed to present a rational chance of success, must, we apprehend, prohibit the surgeon from frequently having recourse to it. It has also been fairly urged by M. Boyer, M. Pelletan, Dr. Cheyne, and other authorities, that, as the exudation extends through the ramifications of the trachea, and probably even through the lungs, there is but little hope of benefit from the operation.

*Laryngitis œdematosa.*—Mr. Porter very truly remarks, that it is no inconsiderable proof of the defective state of pathological knowledge in general, that a disease so very fatal as this, and certainly not so unfrequent as to justify ignorance on the subject, should have been, so recently as the year 1808, viewed with all the interest of a new discovery. It is probable that before this period the disease was mistaken for croup.

"The seat of this affection is more in the cellular tissue, connecting the mucous membrane to the adjacent parts, than in the membrane itself, although this latter structure is very frequently found to have been inflamed. This tissue is reticular, and the effect of inflammation upon it is to cause an effusion of serous fluid within the cells, and thus to create, by approximating the sides of the rima glottidis, a directly mechanical obstruction to the passage of the air to the lungs. It is evident now that the danger of such an affection must be proportioned to the quantity of effusion that takes place, and the rapidity with which it is formed, so that a patient may be quickly suffocated by the complete closure of the rima, or he may be left to struggle during three or four days with partially obstructed respiration, and finally perish of congestion in the lungs and brain. When inflammation of the mucous membrane has accompanied this affection, I cannot find any satisfactory examples of its having extended beyond the larynx, and into the trachea; on the contrary, the chief intensity of disease has been in the epiglottis, which is found red, erect, thickened, and swollen, and during life has been seen to resemble a piece of raw meat." (P. 93.)

This disease is occasionally very insidious in its approach. Mr. Porter has known two instances "of young men who had retired to bed at night without complaining, and were found dead from this affection the next morning." We must refer to the volume itself for the symptomatology of this disease. Although by proper treatment we may check the inflammatory action, we shall not be able to remove the fluid effused in this disease.

"The only manner, then, in which we can reasonably promise safety to our patient, is to procure for him some mode by which respiration can be carried on, other than the larynx which is no longer competent to the performance of its functions, and thus afford time either for the spontaneous subsidence of the disease, or its removal by medical treatment.

"Besides the uncertainty that must prevail as to the precise nature of the morbid action that is going forward in acute laryngitis, and the consequent hazard a practitioner will run of losing his patient whilst he is attempting a treatment that may be unsuccessful, there are many reasons why he should, in the present instance, decide at once on performing bronchotomy. Thus, it allows the organ in which the diseased action is situated to remain in a perfect state of repose. It takes the place of treatment which, besides being injurious from the loss of time, is often in itself positively detrimental. Considered as a wound, it adds nothing to the patient's danger; and as the relief it affords is, at least for some time, complete, it imparts confidence to the surgeon, and allows him more leisure to examine the symptoms, and adapt his remedies accordingly." (P. 100.)

It must not be forgotten, that if effusion into the submucous tissue has taken place, every moment suffered to elapse before an artificial opening is established, must be pregnant with danger. "If, indeed, the operation be not early performed, it had much better be let alone altogether."

*Chronic Cynanche Laryngea.*—Under this name may be included all those affections of the larynx which materially interfere with respiration, but which commence so insidiously, and proceed so slowly, as often to produce an incurable disease before the patient's attention is aroused to the perilous nature of his condition. "Very generally the result of these chronic affections is a morbid alteration of structure that can never be removed; but it is also to be recollected that the symptoms will not be sufficient to mark a distinction between diseases of the larynx that are curable, and those which are not."

Our own practice has furnished us with two or three opportunities of verifying the following remark of our author:

"There is an exceedingly curious circumstance connected with laryngeal pathology, which is, that all the symptoms of difficult respiration may exist, and even prove fatal, whilst the internal structural derangement is either not observable, or so trifling as scarcely to account for the severity of the affection. The cellular substance *external* to the larynx and trachea is sometimes the seat of inflammation and suppuration; and I am in possession of a case in which no diseased appearances were found after death, except purulent matter surrounding the external surface of the entire windpipe. A nearly similar case is mentioned in the eleventh vol. of the Edinburgh Journal, excepting that in this latter there was a laryngeal disease also, but scarcely sufficient to account for the unfortunate result. It is not easy to explain how such external affection can produce internal functional disease, unless, perhaps, by attributing it to the effects of spasm; but the fact is exceedingly interesting, as it shows the difficulties that surround these forms of morbid action, and how very attentively they must be studied in order to form a rational conception of the nature of each individual case." (P. 116.)

In chronic cynanche laryngea, Mr. Porter is of opinion that the operation of bronchotomy will usually be successful if not too long delayed. He has once himself performed it with success. In laryngeal diseases, mercury is generally found very serviceable. If the symptoms are severe, the author has been in the habit of giving calomel in ten-grain doses four times a-day. "As soon as the specific effects of the medicine become developed, the disease begins to decline, and it seldom requires more than a week or ten days to render the cure complete."

Some interesting observations are made upon the subject of *Phthisis Laryngea.*

*Foreign bodies in the Larynx and Trachea.*—From this accident the utmost distress, and generally the rapid destruction of the patient, follow, if the foreign body cannot be expelled by the efforts of nature, or surgical assistance be not promptly afforded.

"When the accident has occurred to an adult, and he is aware of its nature, the surgeon has only to set about affording him the necessary relief; but it does not always happen that persons, even of advanced age, can tell distinctly what it is that occasions the unpleasant symptoms with which they find themselves attacked so suddenly. In times of hurry, terror, or confusion, or under any circumstance that will induce a man to make a hasty or a violent inspiration, a foreign body held incautiously in the mouth may glide imperceptibly into the larynx, and the first notice of the accident be the terrible cough it occasions. Many persons are so addicted to the silly trick of putting extraneous substances into their mouths, and retaining them there, that they are absolutely unconscious of their presence, and the mischief may so unwittingly occur, that even an adult may be unable to afford any satisfactory reasons for the symptoms under which he labours. But it is rarely amongst this class of persons that foreign bodies gain admittance into the windpipe, but rather amongst children, many of whom are unable to explain its nature or its cause, even if they were aware of it, and who moreover are so liable to be influenced by sudden fright, and the dread of surgical interference, that they would be disposed to conceal it, if it was possible." (P. 178.)

Within the last year, two cases have come to the author's knowledge, in both of which, bronchotomy was performed with success. The following case shows the uncertainty with which such accidents are sometimes attended:

"In the month of July, 1822, I was requested to examine the body of a child that had died under circumstances which threw considerable doubt on the nature of the case. On the evening of the day but one preceding her death, she had been playing in the street, when the shaft of a gig or jaunting-car, in pretty rapid motion, struck her, and the by-standers declared that the wheel had passed over her breast. She was taken up, and in a few minutes so far recovered as to be able to walk home without assistance; but from the instant the accident occurred her breathing became croupy. She was, at irregular intervals, teased with an exceedingly distressing cough, and suffered greatly from incessant restlessness, not being able to remain for any time, however short, in one position. This state continued until five o'clock in the morning of the third day, (about thirty-eight hours,) when having arisen for a moment, to allow her father, who had been up all night, room to lie down in the bed, she was seized with a paroxysm of convulsive cough, flung her head in agony on the pillow, and was dead in an instant.

"On examining the body, the thorax was the first part to attract attention, from the circumstance of its having been said that the wheel of a car had gone over it: however, on minute inspection, not a single trace either of injury or disease could be discovered. The viscera of the abdomen were also healthy, and altogether I had never examined a subject so entirely free from every morbid appearance. The trachea was next to be inspected; and in it, or rather in the larynx, was found part of an almond-shell, its rough and broken edge entangled in the rima glottidis, and placed in such a manner that it effectually closed up the aperture for the transmission of air. The nature of the case was now made evident. The child had the fragment of shell in her mouth at the time she was struck, and, either from the fright or the shock, had unconsciously swallowed it, and it passed into the trachea. It was rough and irregular on its surface and edges, and its presence must have occasioned great irritation. There were frequent paroxysms of coughing; and in one of these the edge of the shell was thrown into the rima, choked it up, and the child died from direct suffocation." (P. 181.)

In this case, the existence of a foreign body in the larynx and trachea had never been suspected, nor did the symptoms warrant such a supposition.

The whole chapter upon the subject of foreign bodies in the larynx and trachea, and the section upon wounds of the same parts, demand especial attention. They are cases in which the utmost decision is required. The loss of a little time will frequently be the loss of life to the patient.

The space we have devoted to Mr. Porter's volume is a sufficient proof that we think very favourably of its merit. Every practitioner must be interested in acquiring the best information upon the important subjects he has discussed, and the work will no doubt meet with very general attention.

---

*Medico-Chirurgical Transactions, published by the Medical and Chirurgical Society of London.* Vol. XIII.—8vo. pp. 580; with Plates. Longman and Co. London, 1827.

We have just received a copy of the above volume, and have barely time to introduce it to our readers. It contains several good papers, and some of indifferent merit; but it is chiefly interesting as a proof of the renewed interest which the profession takes in the Society, which had begun, it must be acknowledged, to fall into alarming decay. The plan which we purpose to adopt on this occasion is similar to that which we have previously followed with regard to the Dublin Hospital Reports: we shall not attempt to analyse the contents of a volume embracing such multifarious subjects, but shall embody in our Collectanea extracts from the most interesting papers. Some of these will be found in the present Number, and others will be given hereafter. As a general character, we would say that the volume is inferior to some of the early ones, but better than many of the more recent: the plates are particularly good. In another part of the present Number, we have alluded more particularly to the improved prospects of the Society, and to the hope afforded of a volume of Transactions being published at regular periods.

